# A hybrid gene selection algorithm based on interaction information for microarray-based cancer classification

**DOI:** 10.1371/journal.pone.0212333

**Published:** 2019-02-15

**Authors:** Songyot Nakariyakul

**Affiliations:** Department of Electrical and Computer Engineering, Thammasat University, Khlongluang, Pathumthani, Thailand; Institute for Bioscience and Biotechnology Research, ITALY

## Abstract

We address gene selection and machine learning methods for cancer classification using microarray gene expression data. Due to the high dimensionality of microarray data, traditional gene selection algorithms are filter-based, focusing on intrinsic properties of the data such as distance, dependency, and correlation. These methods are fast but select far too many genes to use for the classification task. In this work, we present a new hybrid filter-wrapper gene subset selection algorithm that is an improved modification of our prior algorithm. Our proposed method employs interaction information to rank candidate genes to add into a gene subset. It then conditionally adds one gene at a time into the current subset and verifies whether the resultant subset improves the classification performance significantly. Only significant genes are selected, and the candidate gene list is updated every time a gene is added to the subset. Thus, our gene selection algorithm is very dynamic. Experimental results on ten public cancer microarray data sets show that our method consistently outperforms prior gene selection algorithms in terms of classification accuracy, while requiring a small number of selected genes.

## Introduction

In recent years, analysis of microarray gene expression data has become an important tool for providing clinical decision support in cancer diagnosis [1,2], for genes have been found to be expressed at significantly different levels in normal and cancer cells. One of the main applications of microarrays in medicine is class prediction [3], which is to identify the class membership of a sample based on its gene expression profile. The process involves the construction of a statistical classifier that learns from the training set data and predicts the class membership of the test samples. However, microarray data contain the expression of thousands of genes, while there are a limited number of samples available for analysis. This curse of dimensionality presents a challenging problem for class prediction, for it often results in high generalization error. One effective solution to alleviate the problem is to perform gene selection to reduce the dimensionality of the microarray data [4,5].

Gene selection is to select a highly discriminative subset of the original genes for use in model construction and gene expression analysis. Based on how they select genes and utilize the learning classifier, gene selection algorithms [6] fall into three categories, namely filter, wrapper, and embedded methods. Filter methods [7–9] select subsets without any knowledge of a learning classifier and thus evaluate subsets based on the intrinsic properties of the data such as distance, dependency, and correlation. They are relatively fast and unbiased in favor of a specific classifier. On the other hand, wrapper methods [10,11] use the performance of a classifier as the criterion function to assess the quality of a selected subset. The wrapper method generally achieves better classification performance than the filter method for the same number of selected genes, but it is also more time-consuming. Some hybrids of filter and wrapper methods are also introduced in the literature [12]. Embedded methods [13,14] perform the search for an optimal subset by interacting with the unique structure of a specific classifier. Unlike wrapper methods, they embed gene selection with classifier construction during learning. They are faster than wrapper methods but are specific to the classifier.

Many gene selection techniques in the literature are filter-based because they are fast and computationally efficient. The fast correlation-based filter (FCBF) algorithm developed by Yu and Liu [15] ranks genes in descending order according to their correlation values with the class. It then adopts correlation measure to remove genes that are redundant to the top ranked genes. The minimal-redundancy-maximal-relevance (mRMR) method [7] selects a gene subset based on mutual information. An information-theoretic criterion is proposed to choose genes that are irredundant to already selected genes and highly correlated with the class. On the other hand, although they are time-consuming, wrapper-based gene selection algorithms have been studied because they are capable of giving high classification accuracy. Inza et al. [16] employed sequential search algorithm on two public microarray data sets. Like FCBF, the best incremental ranked subset (BIRS) algorithm [10] begins by ranking genes according to their individual discriminative power. The search then proceeds from the best to the worst ranked feature, and a feature is selected if adding it to the currently selected feature subset improves the accuracy significantly. The paired *t*-test statistical significance was used as the criterion for gene addition. Wrapper methods are computationally intensive, since they require a classification model to assess the performance of each subset during search. Furthermore, they may be prone to overfitting. To lower time consumption, Guyon et al. [13] proposed an embedded method utilizing support vector machines (SVM) and recursive feature elimination (RFE) called the SVM-RFE method for cancer classification. The authors used the weights of the SVM classifier to produce a feature ranking and iteratively removed the least important genes during training. Experimental results on two microarray data sets showed that SVM-RFE outperformed a prior method based on gene correlation with the classes. Variants of the SVM-RFE method [14] are also available in the literature.

Recently, we proposed a hybrid of filter and wrapper methods called the interaction information-guided incremental selection (IGIS) algorithm [17] for high-dimensional feature selection. The IGIS method attempts to find a subset of features that interact with one another and are relevant with the class, since some weak individual features can provide strong discriminative power when combined together. However, we found that IGIS selected many more features on average than prior wrapper and hybrid methods and that the search terminated too early. For gene selection applications, that many genes are selected by a gene selection algorithm is not preferable, since it becomes difficult to analyze the results. Thus, the IGIS method needs to be improved.

This work focuses on developing a new gene selection and machine learning method to accurately predict cancer outcomes using microarray data. We present an improved interaction information-guided incremental selection (IGIS+) algorithm which is an extension of the original IGIS algorithm. Our aim is to reduce the number of selected genes and to improve the classification accuracy of the original method. We have thoroughly revised three major aspects of the original work and applied it on microarray data sets for cancer classification. The three modifications include (1) selecting a better first selected gene with high discriminative power, (2) introducing a different significance criterion for adding a new gene to the subset, and (3) proposing new stopping criteria to allow more thorough search. These modifications are significant and greatly improve the performance of the original IGIS algorithm. We test our proposed IGIS+ algorithm on ten cancer microarray data sets using the K-nearest-neighbor (KNN) and the decision tree classifiers and compare the results with those of the original IGIS algorithm. These experimental results are new. They confirm that our gene selection method consistently yields higher classification accuracy than prior state-of-the-art wrapper and hybrid algorithms do and requires a small number of selected genes.

## Materials and methods

### The original IGIS algorithm

The IGIS method [17] is a hybrid method that selects a subset of features using interaction information. First, it computes a list of candidate features that have strong interaction information with current selected features. Next, it sequentially adds candidate features one at a time to the selected subset and calculates the performance of the resultant subset. Only a feature that improves the accuracy significantly when added to the current subset is selected. The algorithm re-ranks the candidate features every time the selected feature set is updated. In addition, IGIS employs early stopping to prevent overfitting and to accelerate the speed.

For a feature set *F* with *N* features, *F* = {*X*_1_, *X*_2_, …, *X*_*N*_}, with the target class *C*, IGIS selects a subset of feature *S*, where *S* ⊆ *F*, that aims to maximize the classification accuracy. The target class *C* can be either binary or multiclass. IGIS can be summarized as follows.

**Step 1 (Initialization)**: Let the selected feature set *S* be an empty set. The first selected feature *X*_*k*_ from the full set *F* is one that gives the largest information gain [7].
  $$X_{k}=\text{arg}\;\underset{X_{j}\in F}{\text{max}}\left[I(X_{j};C)\right],$$ (1)
  where *I*(*X*_*j*_; *C*) measures the information about *C* provided by *X*_*j*_. *X*_*k*_ is added to the set *S* and then removed from the set *F*.
**Step 2 (Filter approach)**: The next candidate feature *X*_*d*_ to be added to *S* is one that maximizes the joint mutual information (JMI) criterion [18]:
  $$X_{d}=\text{arg}\;\underset{X_{j}\in F}{\text{max}}\left[I(X_{j};C)+\frac{1}{\left|S\right|}\sum_{X_{i}\in S}I(X_{j};X_{i};C)\right],$$ (2)
  where *I*(*X*_*j*_; *X*_*i*_; *C*) is the interaction information [19] between *X*_*j*_, *X*_*i*_, and class *C*. *X*_*d*_ is thus the candidate feature that has the largest value of information gain and average interaction information with currently selected features and class *C*.
**Step 3 (Wrapper approach)**: The candidate feature *X*_*d*_ is conditionally added to the current set *S*, and a *k*-fold cross-validation is used to calculate the classification accuracy of the training set using the resultant subset. If there is a statistical difference of the classification accuracy between before and after adding *X*_*d*_ to the set *S* measured by a Student’s paired right-tailed *t*-test (at 0.1 level), go to *step* 4. Otherwise, *X*_*d*_ is not selected and then removed from the set *F*. If *F* is empty, terminate the algorithm. Otherwise, go to *step* 2.
**Step 4 (Incremental selection)**: The classification accuracy for the validation set with the subset *S* ∪ *X*_*d*_ using a given classifier is computed. If it does not decrease significantly (using a Student’s paired left-tailed *t*-test at 0.1 level), permanently add *X*_*d*_ into the set *S* and then remove *X*_*d*_ from the set *F*, update the accuracy rates for the training and validation sets, and go to *step* 2. Otherwise, *X*_*d*_ is not selected and the search terminates.

To summarize, since the JMI criterion in Eq (2) may become inaccurate for high-dimensional data sets, IGIS uses a wrapper approach in step 3 to verify whether or not the candidate feature is useful by adding it to the currently selected feature set and computing the classification accuracy using a given classifier. Only a feature that improves the accuracy significantly when added to the selected subset is selected. Step 4 implements early stopping to prevent overfitting and poor generalization and terminates as soon as the validation set accuracy rate decreases. The IGIS algorithm yields higher classification rates than prior hybrid and wrapper methods for high-dimensional data sets but also selects more features for classification [17].

### The improved IGIS (IGIS+) algorithm

We now discuss the major drawbacks of the original IGIS method. First, the algorithm selects more features (genes) on average than prior wrapper or hybrid algorithms do. Second, the paired *t*-test for significance testing requires that the differences between the two groups are normally distributed. However, due to the small sample size, the data may violate the normal assumption, and the *t*-test can be invalid. Third, IGIS terminates very early in some cases due to some outlier genes that overfit the training data and incur high error rates on the validation set. Thus, many good genes may never be evaluated by a given classifier because of early termination.

We propose three major modifications to improve the original IGIS algorithm for gene selection as follows.

#### 1. A better first selected gene

To reduce the number of selected genes, we need to select the best first selected gene. Since the best first selected gene will yield a high performance rate, it is very likely that only a small number of features will be needed to be added to the selected subset after the first gene addition. The first modification to the original IGIS method is to select the first gene that gives the highest training set accuracy rate, not one that gives the largest mutual information between the feature and the class target *C*. That is, the first selected gene, *X*_*k*_, becomes

$$X_{k}=\text{arg}\;\underset{X_{i}\in F}{\text{max}}(\text{Acc}(X_{i})),$$ (3)

where Acc(*X*_*i*_) is the training set accuracy rate obtained using only gene *X*_*i*_ for classification.

#### 2. A different significance criterion

When the sample size *k* (*k* = 4 from fourfold cross-validation in our experiment) is small, the paired *t*-test to compare two accuracy averages can be invalid because the data may violate the normal assumption. Thus, in the IGIS+ method, Cohen’s *d* effect size [20,21], rather than the paired *t*-test, is used to measure the standardized difference between two means of classification accuracy (before and after gene addition). Cohen’s *d* estimates the magnitude of an effect relative to the variability in the population. It is defined [20] as

$$d=\frac{\mu_{t}-\mu_{c}}{s_{\text{pooled}}},$$ (4)

where

$$s_{\text{pooled}}=\sqrt{\frac{(n_{t}-1)s_{t}{}^{2}+(n_{c}-1)s_{c}{}^{2}}{n_{t}+n_{c}}}.$$ (5)

*μ*, *s*, and *n* are the mean, the standard deviation, and the number of cases, respectively. Subscripts *t* and *c* refer to the treatment and control conditions, respectively. In our cases, *μ*_*c*_ and *μ*_*t*_ are the means of *k* classification accuracy rates obtained from *k*-fold cross-validation before and after gene addition, respectively, and *n*_*t*_ and *n*_*c*_ are both equal to *k*. As noted in [21], effect sizes *d* of 0.15 are small, 0.40 are medium, and 0.75 are large. Cohen [20] stated that medium effect sizes “represent an effect likely to be visible to the naked eye of a careful observer.” A gene is added to the selected gene subset if and only if adding it provides a medium or larger positive effect size on the *training* set accuracy rates and a small or larger positive effect size on the *validation* set accuracy rates.

#### 3. New stopping criteria

To prevent the search from terminating prematurely, we propose the following modification. If adding gene *X*_*d*_ to the selected gene subset gives a medium or larger positive effect size on the training set accuracy rates but does not give a small or larger positive effect size on the validation set accuracy rates, *X*_*d*_ is discarded and the algorithm continues. The search terminates when one of these stopping criteria occurs: (a) all genes are explored by a given classifier; (b) all unexplored genes give negative JMI criterion values in Eq (2) (i.e., all unexplored genes are redundant); or (c) the average accuracy rate for the training or validation set reaches 100% (i.e., optimal performance is obtained). As a result, the IGIS+ algorithm can be more computationally expensive than the original IGIS method, since more genes are expected to be evaluated by the classifier. We expect that a more thorough search will provide a better search result.

The IGIS+ algorithm is designed to select a small number of genes and to provide high classification performance by choosing a good first gene, employing a valid significance criterion, and performing a thorough search. The pseudocode of the IGIS+ algorithm is as follows:

**IGIS+ (improved interaction information-guided incremental selection) algorithm**

**Input**: A data matrix of size *M* × *N*, where *M* is the number of samples and *N* is the number of genes, a target class *C* of size *M* × 1, a full gene set *F* of *N* genes, and a given classifier

**Output**: The selected gene subset *S*

1 Select the first gene Xk using Eq (3) and initialize set S = {Xk}

2 Remove Xk from set F

3 Compute k training set accuracy rates, BestAcctrain, with set S using k-fold cross-validation

4 Compute k validation set accuracy rates, BestAccval, with set S using k-fold cross-validation

5 while the stopping criterion is not true

6  Select the candidate gene Xd using Eq (2)

7  Remove Xd from set F

8  Stmp = S ∪ {Xd}

9  Compute k training set accuracy rates, Acctrain, with set Stmp using k-fold cross-validation

10  if Cohen’s d effect size between Acctrain and BestAcctrain is greater than or equal to 0.40

11   Compute k validation set accuracy rates, Accval, with set Stmp using k-fold cross-validation

12   if Cohen’s d effect size between Accval and BestAccval is greater than or equal to 0.15

13    BestAcctrain = Acctrain

14    BestAccval = Accval

15    S = Stmp

16   end if

17  end if

18 end while

19 Output set S

The MATLAB codes of the IGIS+ algorithm are available from https://figshare.com/projects/Gene_selection_for_microarray-based_cancer_classification/56858.

### Microarray data sets

Table 1 details ten public microarray data sets [22–30] used in this work. These data sets represent a broad range of cancer-related two-class and multiclass classification problems. They are very high-dimensional, for the number of genes in each data set is large (from 2000 to 15,154) compared to the number of samples (from 60 to 253). The ten data sets are obtained in the formats provided by the original authors. For example, the colon data set is unprocessed, while the ALL-AML data set is transformed to base 10 logarithms. We then perform a simple rescaling for each data set, so that each gene value in the data set is between 0 and 1 in order to avoid bias in a classifier. The datasets generated and/or analyzed during the current study are formatted and saved in MAT-files for use in MATLAB and also in tab-delimited format for use in other programming languages. They are publicly available at https://figshare.com/projects/Gene_selection_for_microarray-based_cancer_classification/56858.

**Table 1:** Number of genes, samples, and class cardinality in each cancer-related microarray data set.

Data set	Description	Samples	Genes	Classes	Reference
Colon tumor	Colon cancer and normal parts	60	2000	2	Alon et al. [23]
SRBCT	4 types of the small, round blue-cell tumors (SRBCTs)	83	2308	4	Khan et al. [24]
Lymphoma	3 prevalent adult lymphoid malignancies	62	4026	3	Alizadeh et al. [25]
CNS	Patient outcomes for central nervous system (CNS) embryonal tumors	60	7129	2	Pomery et al. [26]
ALL-AML	Acute lymphoblastic leukemia (ALL) and acute myelogenous leukemia (AML)	72	7129	2	Golub et al. [27]
ALL-AML-3	AML, ALL B-cell, and ALL T-cell	72	7129	3	Golub et al. [27]
ALL-AML-4	AML bone marrow, AML peripheral blood, ALL B-cell and ALL T-cell	72	7129	4	Golub et al. [27]
MLL	AML, ALL, and mixed-lineage leukemia (MLL)	72	12,582	3	Armstrong et al. [28]
Lung cancer	4 types of lung tumors and normal lung	203	12,600	5	Bhattacharjee et al. [29]
Ovarian cancer	Normal and ovarian cancers	253	15,154	2	Petricoin et al. [30]

### Experimental design

We compare the performance of the IGIS+ algorithm with those of the BIRS algorithm, the BIRS method with re-ranking mechanism [31] and the original IGIS algorithm. These methods have been shown to outperform prior filter-based gene selection algorithms. As noted earlier, the BIRS algorithm is a well-known wrapper method that selects a few genes for classification and outperforms traditional gene selection algorithms. BIRS with the re-ranking mechanism, denoted by BIRS^R^, employs conditional mutual information maximization (CMIM) criterion to rank a block of best *B* (*B* = 30) genes and incrementally added genes one at a time to the selected subset similar to BIRS. If new genes are added to the current subset, a new set of *B* ranked genes are computed, and the process is repeated. If there is no new gene added to the subset when evaluating a block of best *B* genes, the process terminates. To calculate the information-theoretic criterion in BIRS^R^, IGIS, and IGIS+, each gene value is discretized into three states at the positions *μ* ± *σ*, where *μ* is the mean value and *σ* is the standard deviation; it becomes −1 if the value is less than *μ* − *σ*, +1 if the value is larger than *μ + σ*, and 0 otherwise. We only use discretized data to compute the criterion value for the gene selection. After the candidate gene is selected, we feed un-discretized data with the resultant subset into a classifier to obtain the exact accuracy rates.

**Fig 1:**
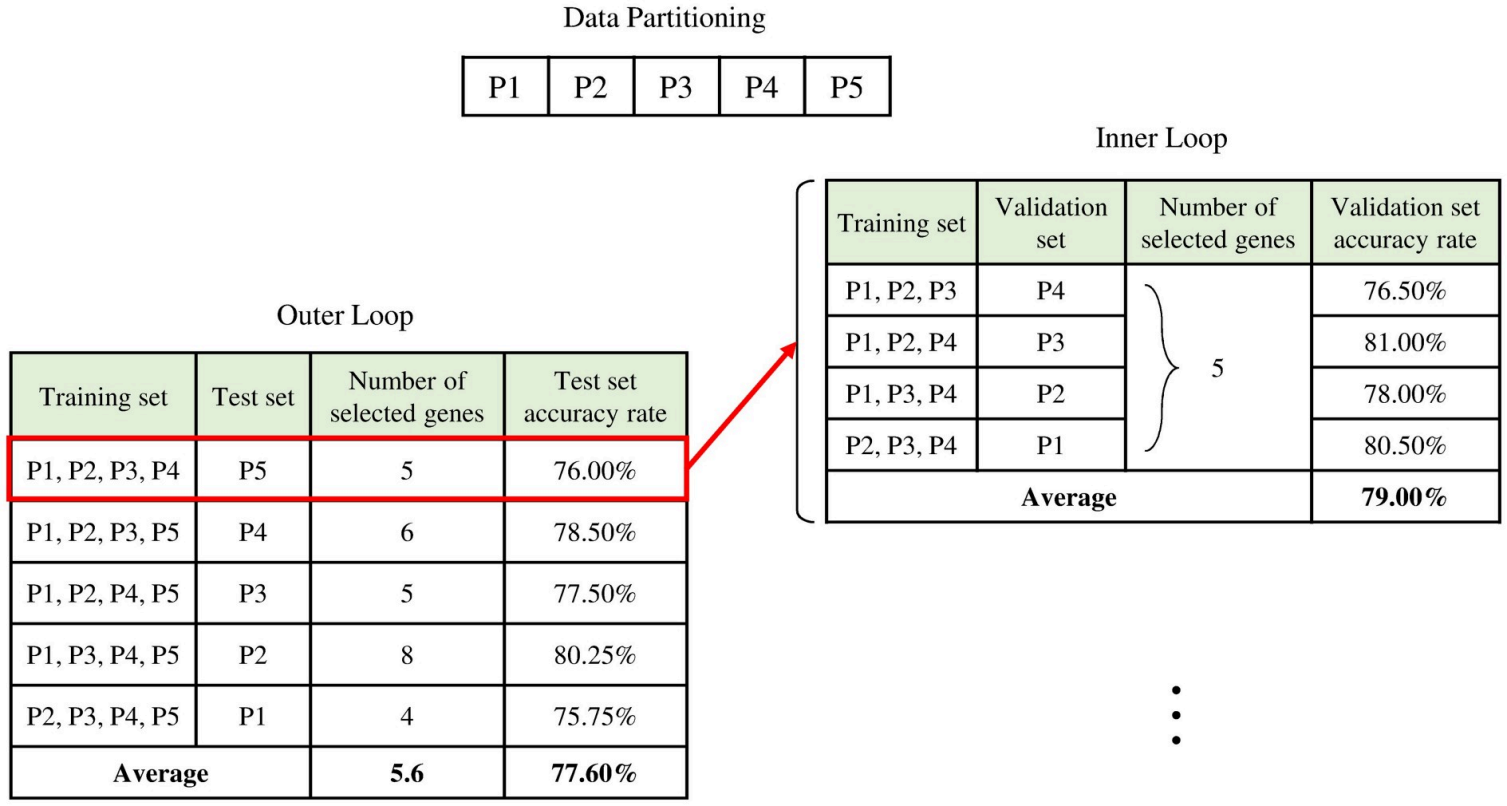
Simplified example of a nested stratified fivefold cross-validation.

Due to a limited number of samples in each data set, we perform nested cross-validation based on two loops for performance estimates. In the outer loop, a stratified fivefold cross-validation is used to assess the overall accuracy. A fourfold cross-validation (*k* = 4) in the inner loop is used to determine the best number of genes selected by the gene selection algorithm for use. Fig 1 illustrates an example of nested stratified fivefold cross-validation used in our experiment. We partition data into five mutually exclusive sets P1 to P5. For the outer loop, a set of four partitions is used for training, and the remaining partition is used for testing. The gene selection algorithm performs cross-validation in the inner loop to determine the best number of genes for use in the outer loop. For our example, when the four partitions (P1, P2, P3, and P4) are trained by the gene selection algorithm in a cross-validated fashion, we assume that five genes are selected, for they yield the best average validation set accuracy rate over four validation sets. To optimize the speed of the IGIS+ algorithm, we use the four partitions to select the first gene with the highest accuracy rate. Then, the fourfold cross-validation in the inner loop is employed to add more genes into the selected subset that improve the performance significantly. In the outer loop, the first training set (P1, P2, P3, and P4) with the selected five genes is used to compute the test set accuracy rate on the test set P5. Thus, the test set P5 is unseen and not trained by the gene selection algorithm. For fair comparisons, all four gene selection algorithms use the same partitions for training and testing. The average performance of fivefold cross-validation in the outer loop is recorded on each run. We perform ten runs for each gene selection algorithm on each data set and report the average results of these ten runs. All codes are implemented in MATLAB, and all experiments are run on an Intel Core i5 computer with 16 GB of RAM.

We consider using two classifiers in our work: the KNN classifier with K = 3 and the CART decision tree classifier. The KNN classifier assigns an object to the class by a majority vote of its K nearest neighbors. We choose K = 3 because we feel that it provides low bias and acceptable variance. A decision tree is a tree where each decision node represents a decision rule and each leaf node is a classification outcome. User-defined parameters are set as default values in MATLAB R2016b. We choose these two classifiers for their simplicity and speed.

To measure the performance of the gene selection algorithms, we use classification accuracy (Acc), which is the percentage of samples that are assigned to the correct class.

$$\text{Acc}=\frac{\text{TP}+\text{TN}}{\text{TP}+\text{TN}+\text{FP}+\text{FN}},$$ (6)

where TP (true positives) and TN (true negatives) are the numbers of positive and negative samples that are correctly classified. FP (false positives) are the numbers of negative-class samples misclassified as the positive class, and FN (false negatives) are the numbers of positive-class samples misclassified as the negative class. When the data sets are not balanced, other performance metrics should be considered. For our experiments, we measure the F-score because it is resilient to class imbalance. It is defined as follows.

$$\text{F-score}=2\cdot \frac{\text{precision}\cdot \text{recall}}{\text{precision}+\text{recall}},$$ (7)

where

$$\text{precision}=\frac{\text{TP}}{\text{TP}+\text{FP}},$$ (8)

and

$$\text{recall}=\frac{\text{TP}}{\text{TP}+\text{FN}}.$$ (9)

For multi-class classification problems, we compute the macro-averaged F-score by averaging the F-score of each individual class. The best F-score is 1 and the lowest possible F-score is 0.

## Results

In this section, we apply our IGIS+ algorithm for gene selection on ten microarray data sets. We compare the performance of our proposed algorithm with prior gene selection algorithms in terms of classification accuracy, the number of selected genes, and the number of required wrapper evaluations. We first report the gene selection results using the KNN classifier and then show those using the CART decision tree classifier.

### Results using the KNN classifier

**Fig 2:**
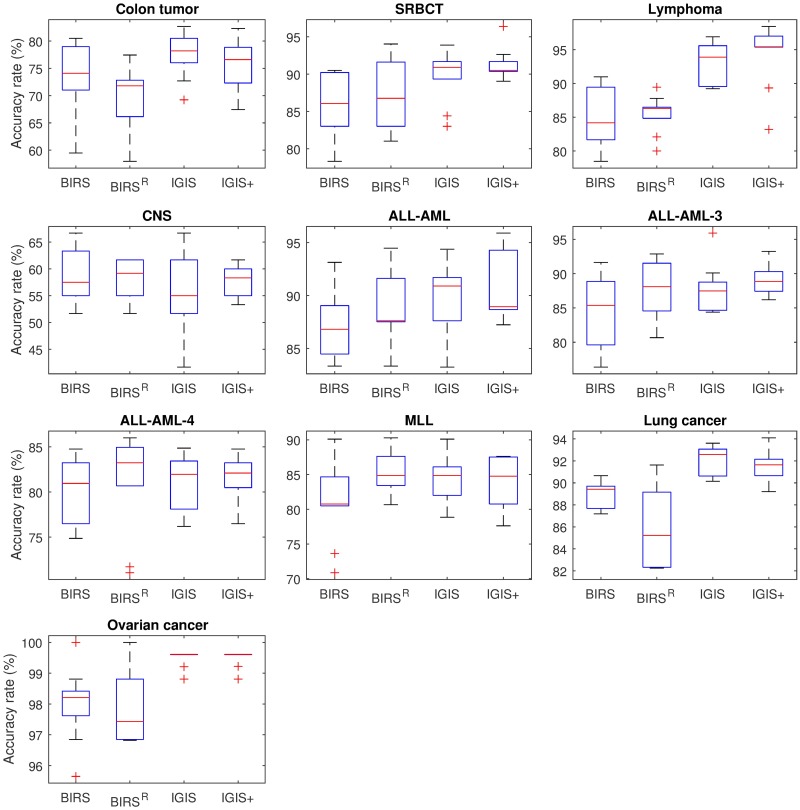
The box plots of the average accuracy rates (for *n* = 10 samples) obtained by BIRS, BIRS^R^, IGIS, and IGIS+ on each microarray data set using the KNN classifier as the classifier. The whiskers are extended to the most extreme data points that are not outliers.

Table 2 summarizes the average test set accuracy rates and the average numbers of selected genes of ten runs of fivefold cross-validation obtained by different methods using the KNN classifier. The best results for each data set are shown in bold. For each data set, the algorithm which obtains the highest accuracy rate ranks first among the four algorithms, while the one with the lowest accuracy rate ranks fourth. For a comparison of the number of selected genes, the algorithm which selects the smallest number of genes ranks first, and the one with the largest number of genes ranks fourth. Average ranks are provided for a fair comparison of the algorithms over ten data sets. Compared with the other methods, the BIRS method is the only method that does not employ any information-theoretic criterion during the search. Using the KNN classifier, BIRS has the highest (worst) average rank of 3.40. The BIRS^R^ algorithm, on the other hand, uses the re-ranking mechanism to rank listed genes. To accelerate the search speed, it terminates when evaluating a blocks of ranked *B* = 30 genes does not improve the result. BIRS^R^ obtains acceptable accuracy rates within a short search time. IGIS and IGIS+ select a group of genes that strongly interact with one another and obtain relatively high accuracy rates. We note that IGIS+ outperforms the original IGIS algorithm on many data sets. This is expected, since IGIS+ improves upon IGIS. Overall, IGIS+ produces the best average accuracy rates on six out of ten data sets and has the lowest (best) average rank of 1.60. Fig 2 shows the box plots of the average accuracy rates of the four gene selection methods on ten microarray data sets using the KNN classifier. It illustrates the spread and differences of ten accuracy averages for each algorithm. Regarding the number of selected genes, a small number of selected genes is preferred. From Table 2, BIRS and BIRS^R^ select the smallest numbers of genes on all data sets at the cost of low accuracy rates. On average, the number of genes selected by IGIS+ is more than 20% smaller than that selected by IGIS because IGIS+ has a better first gene and employs a more meaningful significance criterion than IGIS does.

**Table 2:** The average test set accuracy rates and the average numbers of selected genes of ten runs of fivefold cross-validation obtained by BIRS, BIRS^R^, IGIS, and IGIS+ using the KNN classifier as the classifier.

Data set	Accuracy rate (%)				Number of selected genes			
	BIRS	BIRS^R^	IGIS	IGIS+	BIRS	BIRS^R^	IGIS	IGIS+
Colon tumor	73.64	70.15	**77.47**	76.04	1.6	**1.5**	5.3	3.6
SRBCT	85.54	86.99	90.00	**91.35**	**4.2**	4.5	9.2	8.3
Lymphoma	84.70	85.59	93.33	**94.37**	**1.5**	1.9	3.3	3.0
CNS	**58.67**	58.33	55.17	57.83	2.4	**1.3**	5.7	3.3
ALL-AML	86.98	88.63	89.87	**90.56**	**1.2**	1.4	5.0	3.1
ALL-AML-3	84.78	87.63	87.71	**89.05**	**2.3**	2.6	7.5	4.7
ALL-AML-4	79.84	81.26	81.16	**81.94**	**2.8**	2.9	9.0	6.1
MLL	81.13	**85.35**	84.12	83.91	2.3	**2.2**	7.0	5.6
Lung cancer	89.03	85.71	**92.06**	91.69	7.7	**4.5**	12.4	10.6
Ovarian cancer	97.98	97.78	**99.49**	**99.49**	**1.9**	2.1	2.8	2.9
**Average rank**	3.40	2.90	2.00	**1.60**	**1.40**	1.60	3.90	3.10

**Fig 3:**
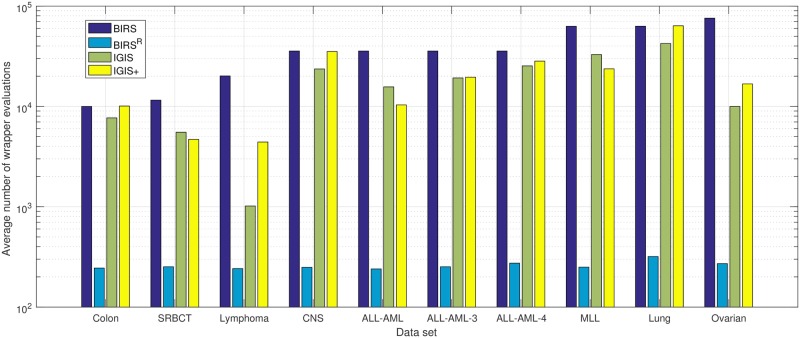
The bar chart of the average numbers of wrapper evaluations of ten runs of fivefold cross-validation required by BIRS, BIRS^R^, IGIS, and IGIS+ on each microarray data set using the KNN classifier as the classifier.

Regarding the computational complexity, we record the number of wrapper evaluations needed by each algorithm because each wrapper evaluation is compute-intensive, for it requires a classification model to assess the performance of each subset. Fig 3 shows the average numbers of wrapper evaluations of ten runs of fivefold cross-validation required by the four gene selection methods using the KNN classifier. A base-10 log scale is used for the Y axis. The higher the average number of wrapper evaluations, the more time-consuming the algorithm. From Fig 3, we see that BIRS^R^ is the fastest algorithm, for only a few blocks of *B* = 30 genes are evaluated before terminating. For a full set of *N* genes, BIRS needs *N* wrapper evaluations to rank all the genes based on their individual discriminative power and performs 4*N* wrapper evaluations using fourfold cross-validation for *N* genes. Thus, the BIRS algorithm requires a fixed number of 5*N* wrapper evaluations, and it is the slowest algorithm. On average, IGIS+ requires more wrapper evaluations than IGIS, since IGIS+ searches more thoroughly than IGIS. We note that even though the ovarian cancer data set has more genes than the lung cancer data set (15,154 versus 12,600), IGIS and IGIS+ need fewer average numbers of wrapper evaluations because the stopping criterion (average training set accuracy rate is 100%) is met very early during the search. In terms of computer time, the computational time depends on many factors including the programmer’s coding style, the used software, the amount of RAM memory, and the processor speed, but it is important to note that the computer time is proportional to the number of wrapper evaluations. To perform one run of fivefold cross-validation on the lung cancer data set (five classes with 12,600 genes), BIRS^R^ takes two minutes, IGIS needs 25 minutes, IGIS+ requires 32 minutes, and BIRS searches for 44 minutes. When we run the four gene selection algorithms on the ovarian cancer data set (two classes with 15,154 genes), BIRS^R^, IGIS, IGIS+ and BIRS take two minutes, ten minutes, 11 minutes, and 80 minutes, respectively. This is expected, since the amount of search time is proportional to the number of wrapper evaluations required by each algorithm as shown on Fig 3.

Many of the microarray data sets are imbalanced, for there are far more negative samples than positive samples. Using accuracy as the performance measure can lead the classification models to be biased towards the majority class. Thus, we consider employing the F-score as the performance metric of the classifier for the gene selection algorithms and compare the results. Table 3 shows the average F-scores and the average numbers of selected genes of ten runs of fivefold cross-validation obtained by four gene selection methods using the KNN classifier. Compared with the accuracy performance, the highly imbalanced data sets such as the ALL-AML-4 and lung cancer data sets yield low F-scores as expected. From Table 4, although IGIS obtains a lower average rank than IGIS+ does regarding the average F-score, both IGIS and IGIS+ algorithms produce the best average F-scores on five out of ten data sets. In terms of the number of selected genes, the number of genes selected by IGIS+ is more than 25% smaller on average than that selected by IGIS. This is consistent with the results obtained using the accuracy performance.

**Table 3:** The average F-scores and the average numbers of selected genes of ten runs of fivefold cross-validation obtained by BIRS, BIRS^R^, IGIS, and IGIS+ using the KNN classifier.

Data set	F-score				Number of selected genes			
	BIRS	BIRS^R^	IGIS	IGIS+	BIRS	BIRS^R^	IGIS	IGIS+
Colon tumor	0.654	0.627	**0.730**	0.709	2.4	**1.4**	5.6	3.6
SRBCT	0.863	0.823	0.912	**0.913**	5.6	**4.2**	8.7	8.7
Lymphoma	0.781	0.744	0.872	**0.879**	2.0	**1.6**	3.6	3.1
CNS	0.519	0.525	**0.529**	0.504	1.7	**1.2**	6.3	3.1
ALL-AML	0.869	0.870	0.880	**0.901**	1.9	**1.5**	5.2	2.9
ALL-AML-3	0.767	0.761	**0.813**	0.804	**2.7**	**2.7**	7.4	5.8
ALL-AML-4	**0.647**	0.632	0.637	0.629	4.8	**3.7**	9.4	4.9
MLL	0.804	**0.838**	0.837	0.828	2.2	**2.1**	7.2	5.5
Lung cancer	0.779	0.769	**0.854**	**0.854**	5.6	**4.7**	12.6	10.8
Ovarian cancer	0.977	0.977	**0.998**	**0.998**	**1.9**	2.0	2.8	2.9
**Average rank**	3.00	3.20	**1.50**	2.00	1.80	**1.10**	3.80	3.10

**Table 4:** The average test set accuracy rates and the average numbers of selected genes of ten runs of fivefold cross-validation obtained by BIRS, BIRS^R^, IGIS, and IGIS+ using the decision tree as the classifier.

Data set	Accuracy rate (%)				Number of selected genes			
	BIRS	BIRS^R^	IGIS	IGIS+	BIRS	BIRS^R^	IGIS	IGIS+
Colon tumor	67.95	70.03	**73.47**	73.15	2.7	**2.6**	4.5	4.5
SRBCT	81.15	78.65	82.42	**83.63**	3.9	**3.3**	5.5	5.3
Lymphoma	85.15	85.15	**90.03**	87.58	2.0	**1.9**	2.4	2.6
CNS	52.50	53.33	52.67	**55.83**	2.9	**2.3**	5.0	5.6
ALL-AML	**85.70**	85.15	85.42	85.40	**1.4**	**1.4**	3.7	2.1
ALL-AML-3	82.51	81.95	80.69	**83.10**	2.7	**2.6**	4.6	3.6
ALL-AML-4	75.69	76.31	74.48	**76.49**	3.2	**2.8**	5.8	4.1
MLL	83.65	83.24	78.34	**83.96**	2.4	**1.8**	3.8	3.6
Lung cancer	83.86	83.50	84.92	**86.73**	4.8	**4.1**	8.0	6.7
Ovarian cancer	95.73	95.73	**97.99**	97.27	**2.2**	**2.2**	2.7	2.7
**Average rank**	2.80	3.10	2.40	**1.50**	1.80	**1.00**	3.60	3.20

### Results using the CART decision tree classifier

**Fig 4:**
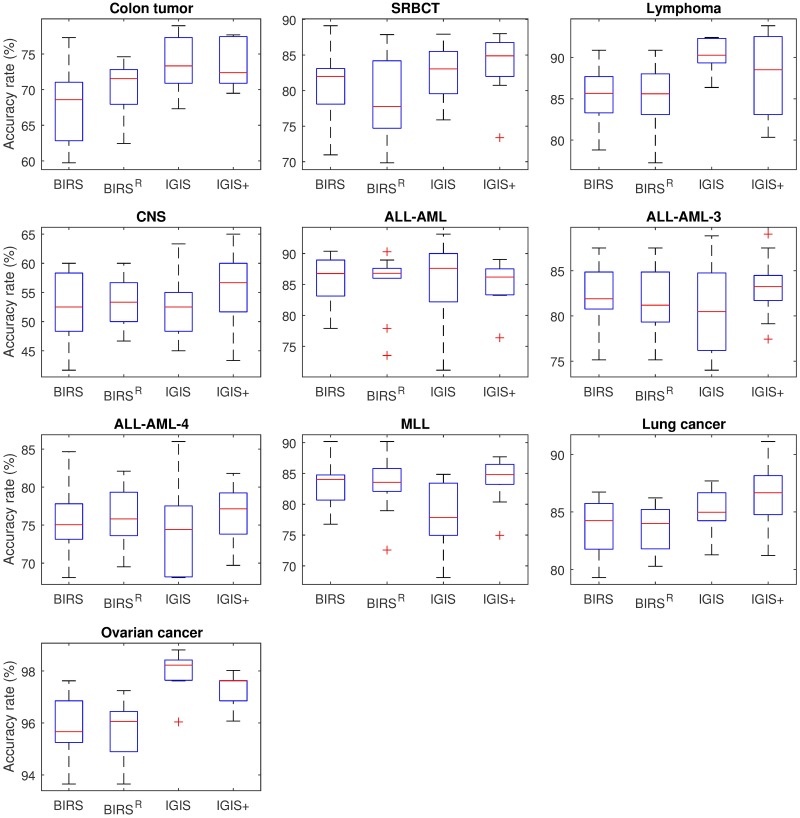
The box plots of the average accuracy rates (for *n* = 10 samples) obtained by BIRS, BIRS^R^, IGIS, and IGIS+ on ten microarray data sets using the decision tree as the classifier.

We now discuss the gene selection results using the decision tree classifier. Table 4 presents the average test set accuracy rates and the average numbers of selected genes of ten runs of fivefold cross-validation obtained by different gene selection methods using the decision tree classifier. We see that using decision tree classifier generally yields lower average accuracy rates than using the KNN classifier. Among the four gene selection algorithms, IGIS+ again has the best average accuracy rates on six out of ten data sets and has the lowest (best) average rank of 1.50, while BIRS^R^ has the highest (worst) average rank. These results confirm that IGIS+ is superior to the BIRS, BIRS^R^, and IGIS methods for the accuracy performance. Fig 4 shows the box plots of the average accuracy rates of the four algorithms on the ten microarray data sets. The box plots illustrate the distributions of ten accuracy averages for each algorithm that are consistent with data in Table 4. Regarding the number of selected genes, Table 4 shows that BIRS^R^ has the lowest average rank, followed by BIRS, IGIS+, and IGIS, respectively. We again see that IGIS+ selects a smaller number of genes on average than IGIS by more than 11%.

**Fig 5:**
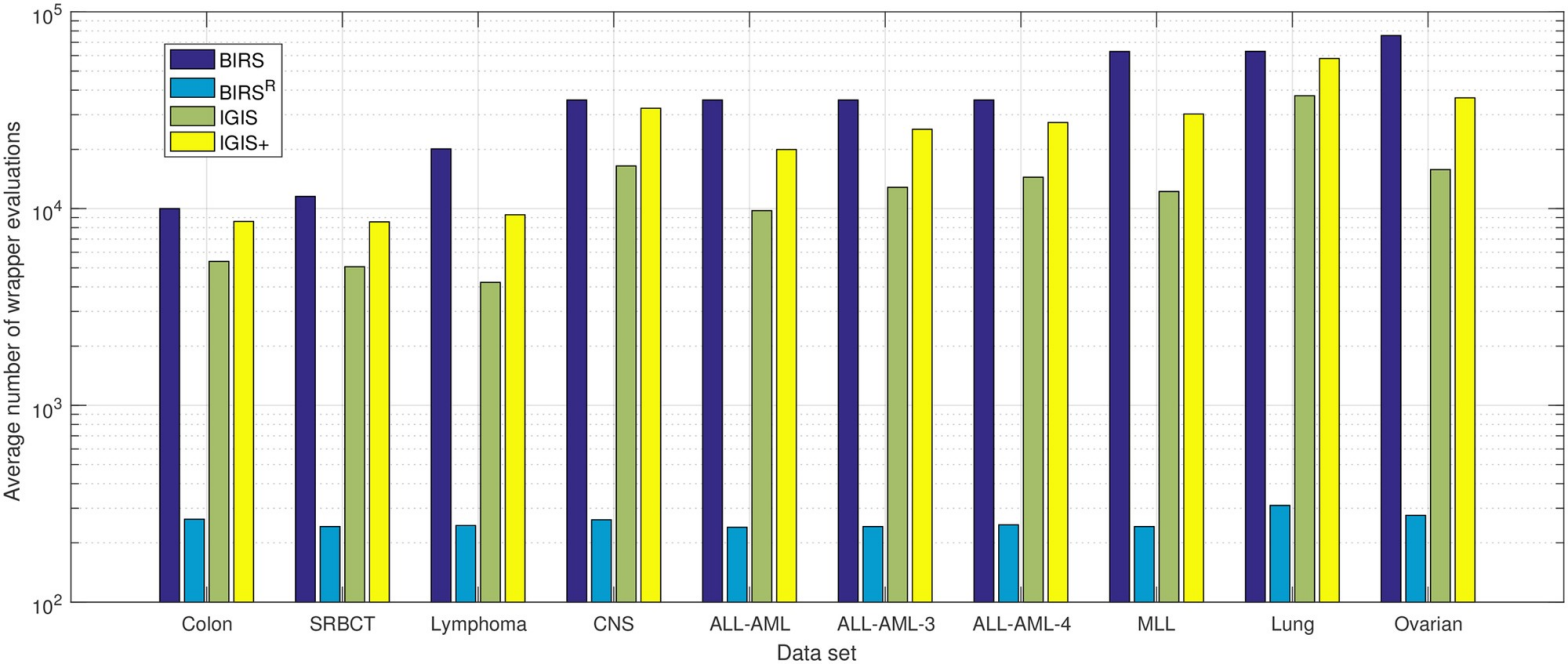
The bar chart of the average numbers of wrapper evaluations of ten runs of fivefold cross-validation required by BIRS, BIRS^R^, IGIS, and IGIS+ on each microarray data set using the decision tree classifier.

Fig 5 shows the average numbers of wrapper evaluations of ten runs of fivefold cross-validation required by BIRS, BIRS^R^, IGIS, and IGIS+ using the decision tree classifier. We obtain similar trends as when the KNN classifier is used as the classifier. BIRS^R^ is the fastest algorithm, while BIRS is the slowest one. On average, IGIS+ requires more wrapper evaluations than IGIS, since it searches more thoroughly than IGIS. Regarding computer time, BIRS^R^ takes six minutes, IGIS needs 42 minutes, IGIS+ requires 55 minutes, and BIRS searches for 67 minutes to perform one run of fivefold cross-validation on the lung cancer data set. The decision tree classifier takes a longer time to assess the performance of each subset than the KNN classifier does. Thus, each gene selection algorithm requires a longer computer time using the decision tree classifier even when it needs the same number of wrapper evaluations. To perform one run of fivefold cross-validation on the ovarian cancer data set. BIRS^R^, IGIS, IGIS+ and BIRS take eight minutes, 24 minutes, 41 minutes, and 104 minutes, respectively. Again, the amount of search time is proportional to the number of wrapper evaluations required by each algorithm as shown on Fig 5.

Table 5 summarizes the average F-scores and the average numbers of selected genes of ten runs of fivefold cross-validation obtained by the four algorithms using the CART decision tree classifier. Compared with Table 4, we see similar trends that in terms of the average F-score, IGIS+ has the lowest average rank, followed by IGIS, BIRS and BIRS^R^, respectively. BIRS^R^ selects the smallest number of genes on average, while IGIS needs the largest number of genes among the four algorithms for classification as expected. The experimental results confirm that IGIS+ is superior to IGIS, since IGIS+ provides a higher F-score and requires a smaller number of genes than IGIS does.

**Table 5:** The average F-scores and the average numbers of selected genes of ten runs of fivefold cross-validation obtained by BIRS, BIRS^R^, IGIS, and IGIS+ using the decision tree classifier.

Data set	F-score				Number of selected genes			
	BIRS	BIRS^R^	IGIS	IGIS+	BIRS	BIRS^R^	IGIS	IGIS+
Colon tumor	0.680	0.652	**0.700**	0.697	3.4	**2.5**	4.6	4.6
SRBCT	0.768	0.763	**0.841**	0.821	3.9	**3.3**	5.4	5.6
Lymphoma	0.748	0.714	**0.831**	0.785	1.9	**1.6**	2.4	2.6
CNS	**0.526**	0.493	0.471	0.512	4.3	**2.2**	5.3	5.5
ALL-AML	0.869	0.854	0.822	**0.870**	1.7	**1.4**	3.6	2.1
ALL-AML-3	0.785	**0.801**	0.722	0.793	**2.5**	2.6	4.6	4.2
ALL-AML-4	0.636	0.628	0.604	**0.652**	3.7	**2.9**	5.8	4.1
MLL	**0.825**	**0.825**	0.757	0.814	**1.9**	**1.9**	4.5	3.7
Lung cancer	0.676	0.674	0.706	**0.734**	5.8	**4.6**	8.7	6.1
Ovarian cancer	0.958	0.956	**0.977**	0.972	**2.0**	2.1	2.7	2.7
**Average rank**	2.40	3.10	2.60	**1.80**	1.70	**1.20**	3.50	3.30

### Analysis on selected genes for potential biomarkers

**Fig 6:**
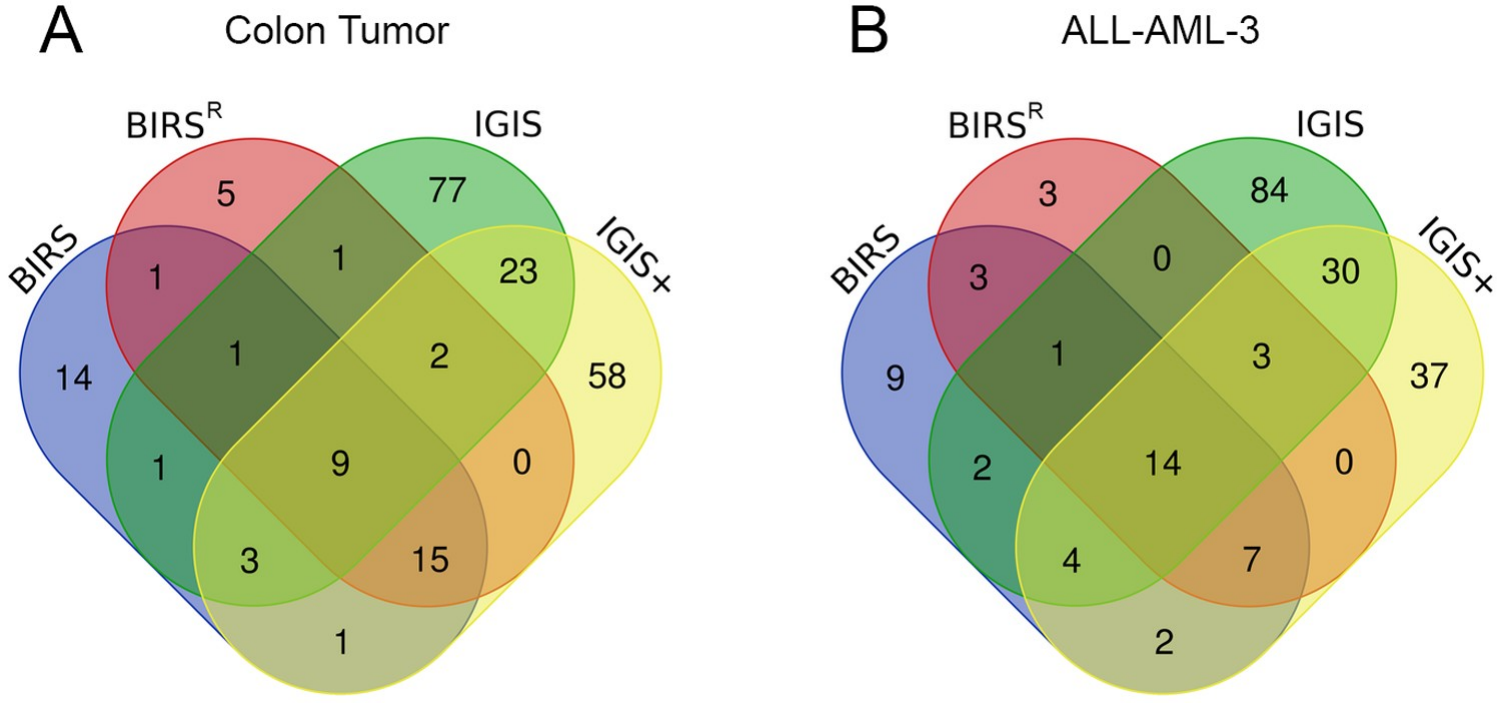
Venn diagrams of genes selected by the four gene selection algorithms using the KNN classifier on (A) the colon tumor data set and (B) the ALL-AML-3 data set.

We now analyze the genes selected by our IGIS+ algorithm for the colon tumor and ALL-AML-3 data sets, for these data sets are extensively studied. Fig 6 shows Venn diagrams of all genes selected by the four gene selection algorithms using the KNN classifier for ten runs of fivefold cross-validation. From Fig 6A, there are nine genes shared by all four algorithms for the colon tumor data set. One of them is J05032 (human aspartyl-tRNA syntetase alpha-2 subunit mRNA). M26383 (human monocyte-derived neutrophil-activating protein (MONAP) mRNA) is selected by BIRS, BIRS^R^, and IGIS+, whereas M63391 (human desmin gene) is chosen by only IGIS and IGIS+. IGIS+ selects 58 unique genes that are not chosen by other three algorithms. One of them is H08393 (collagen alpha 2(XI) chain (Homo sapiens)). These genes listed above are relevant genes for colon tumor detection [32,33]. Within the ten runs of fivefold cross-validation, IGIS+ selects J050032, M26383, and M63391 more than five times.

For the ALL-AML-3 data set, there are 14 genes shared by all four algorithms as seen in Fig 6B. These genes include U05259 (MB-1 gene), X95735 (Zyxin), M23197 (CD33 antigen (differentiation antigen)), and M83652 (PFC properdin P factor, complement). M31523 (TCF3 Transcription factor 3 (E2A immunoglobulin enhancer binding factors E12/E47)) is selected by BIRS, BIRS^R^, and IGIS+, while M84526 (DF D component of complement (adipsin)) is chosen by BIRS^R^, IGIS, and IGIS+. These genes are important biomarkers for identifying the AML and ALL classes [27]. One of the 37 unique genes selected by IGIS+ is M21624 (TCRD T-cell receptor, delta), which is a crucial biomarker for being a direct target of activated NOTCH1 and being upregulated in T-cell ALL [34]. Thus, the IGIS+ algorithm is able to identify more known biomarkers than BIRS, BIRS^R^, and IGIS do. Within the ten runs of fivefold cross-validation, IGIS+ selects M31523 four times, U05259 six times, and X95735 more than eight times.

## Discussion

Our goal in this study is to use gene selection and machine learning methods to accurately predict cancer outcomes using microarray data. We propose a hybrid gene selection named the IGIS+ algorithm that improves upon the original IGIS algorithm. The new modifications of the IGIS+ method include selecting the gene with the highest accuracy rate as the first gene, utilizing Cohen’s *d* effect size as the significance criterion to add a new gene into the selected gene set, and adopting new stopping criteria for extensive search. IGIS+ employs a dynamic search mechanism that is able to find a subset of genes that interact one another and are useful for cancer classification. We compare our proposed algorithm with prior wrapper and hybrid gene selection methods using the KNN and decision tree classifiers. The experimental results demonstrate that using the KNN classifier, the IGIS+ algorithm provides solutions with accuracy rates that equal or exceed those of the BIRS, BIRS^R^, and IGIS algorithms for six out of ten microarray data sets. Furthermore, IGIS+ selects far fewer genes on average than IGIS. Using the decision tree classifier, IGIS+ remains superior to other gene selection algorithms regarding the accuracy rates and needs fewer genes than the original IGIS algorithm as expected. When the F-score is used as the performance metric for the imbalanced data sets, we see similar trends that IGIS+ outperforms IGIS on average using both KNN and decision tree classifiers.
